# Climate Futures for Lizards and Snakes in Western North America May Result in New Species Management Issues

**DOI:** 10.1002/ece3.70379

**Published:** 2024-10-13

**Authors:** David S. Pilliod, Michelle I. Jeffries, Robert S. Arkle, Deanna H. Olson

**Affiliations:** ^1^ U.S. Geological Survey, Forest and Rangeland Ecosystem Science Center Boise Idaho USA; ^2^ Pacific Northwest Research Station U.S. Department of Agriculture, Forest Service Corvallis Oregon USA

**Keywords:** climate change, ectothermy, lizards, niche, North America, potential niche, snakes, squamate, vertebrates

## Abstract

We assessed changes in fundamental climate‐niche space for lizard and snake species in western North America under modeled climate scenarios to inform natural resource managers of possible shifts in species distributions. We generated eight distribution models for each of 130 snake and lizard species in western North America under six time‐by‐climate scenarios. We combined the highest‐performing models per species into a single ensemble model for each scenario. Maps were generated from the ensemble models to depict climate‐niche space for each species and scenario. Patterns of species richness based on climate suitability and niche shifts were calculated from the projections at the scale of the entire study area and individual states and provinces, from Canada to Mexico. Squamate species' climate‐niche space for the recent‐time climate scenario and published known ranges were highly correlated (*r* = 0.81). Overall, reptile climate‐niche space was projected to move northward in the future. Sixty‐eight percent of species were projected to expand their current climate‐niche space rather than to shift, contract, or remain stable. Only 8.5% of species were projected to lose climate‐niche space in the future, and these species primarily occurred in Mexico and the southwestern U.S. We found few species were projected to lose all suitable climate‐niche space at the state or province level, although species were often predicted to occupy novel areas, such as at higher elevations. Most squamate species were projected to increase their climate‐niche space in future climate scenarios. As climate niches move northward, species are predicted to cross administrative borders, resulting in novel conservation issues for local landowners and natural resource agencies. However, information on species dispersal abilities, landscape connectivity, biophysical tolerances, and habitat suitability is needed to contextualize predictions relative to realized future niche expansions.

## Graphical abstract

We assessed changes in fundamental climate‐niche space for lizard and snake species in western North America under modeled climate scenarios to inform natural resource managers of possible shifts in species distributions. Overall, reptile climate‐niche space was projected to move northward in the future. Sixty‐eight percent of species were projected to expand their current climate‐niche space rather than to shift, contract, or remain stable. Only 8.5% of species were projected to lose climate‐niche space in the future, and these species primarily occurred in Mexico and the southwestern U.S.

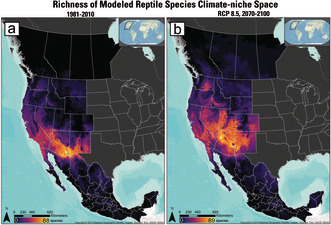

## 1 Introduction

Reptiles are a diverse (Tingley, Meiri, and Chapple 2016; Roll et al. 2017) but relatively understudied group of vertebrates that are likely to respond to changing climates because of their ectothermic physiology, relatively small body size, and limited dispersal capabilities (Ceia‐Hasse et al. 2014; Winter et al. 2016; Diele‐Viegas et al. 2020). In western North America, the highest reptile diversity occurs in the south, where they are well adapted to the hot, dry desert environments (Pilliod et al. 2020; Whiting and Fox 2021). However, reptiles also occupy nearly all ecosystems from Canada to Mexico, apart from some of the highest elevations and latitudes (i.e., reptiles currently do not occur north of 60° north latitude).

As ectotherms, reptiles are strongly associated with, and highly sensitive to, environmental temperatures (Whiting and Fox 2021). Thus, upper elevation limits and northern boundaries of species' ranges are often established because excessive cold or temperature variability limit successful reproduction and survival (Sperry et al. 2010; Hoffmann, Chown, and Clusella‐Trullas 2013), or because climate interacts with other factors (e.g., prey, predators, behavior) to restrict ranges (Wilson and Cooke 2004). Hence, if a reptile species has an innate adaptive capacity, whereby it can either persist‐in‐place or shift‐in‐space (e.g., Thurman et al. 2022) in response to a warming climate, then climate‐based changes to species distributions may be variable but predictable. This enables modeling and mapping of species distributions at broad spatial extents and predicting changes in distributions under future climate change scenarios. Such information can be used by Tribal, state, provincial, and federal natural‐resource managers who are responsible for the management and conservation of these non‐game wildlife species or their habitats. Species‐level assessments of potential shifts in distribution may provide an early warning for agencies managing for squamate species persistence, informing them how they might alter management to account for projected species gains (via colonization, translocation) or losses (local extirpation, extinction).

Like most other vertebrates, reptiles are experiencing elevated extinction risk in the Anthropocene, when human activities have significant effects on the global environment (Böhm, Williams, et al. 2016). Changes in species' distributions and abundance have been attributed to a complex mix of interacting factors (Saha et al. 2018). Global threats to reptiles include habitat loss and degradation, introduced invasive species, pollution, disease, unsustainable use and exploitation, and climate change (Gibbons et al. 2000). Climate change is particularly concerning because reptiles are ectothermic animals with life‐history functions and corresponding diel‐and‐seasonal behaviors that are highly sensitive to changes in environmental temperature; climatic shifts will variably affect populations directly through changing climate regimes and indirectly by creating ecological mismatches, novel or vacant niches, and ecological traps (Cadby et al. 2010; Dell, Pawar, and Savage 2014; Ockendon et al. 2014; Hale and Swearer 2016; Winter et al. 2016). For example, temperature has been documented to alter the phenology of offspring production (e.g., Ockendon et al. 2014), which may shift to a suboptimal time of year that does not align with foraging opportunities (Sinervo et al. 2010; Dell, Pawar, and Savage 2014; Vafidis, Smith, and Thomas 2019) or seasonal weather trends (Wheeler et al. 2015).

Concern for reptiles in western North America is increasing because the region has been susceptible to prolonged drought and is currently experiencing increasing temperatures and altered moisture patterns as the climate changes (Huang et al. 2017; Williams et al. 2020). Recent estimates suggest that at least 20% of all snake and lizard species are highly vulnerable to climate change (Sinervo et al. 2010; Case, Lawler, and Tomasevic 2015; Böhm, Cook, et al. 2016; Jezkova and Wiens 2016; Griffis‐Kyle et al. 2018). Some species may be particularly vulnerable because their close affiliation with specific substrates for thermoregulation and reproduction and relatively limited dispersal abilities can result in small or patchy distributions. For example, a study of 48 *Sceloporus* lizard species across 200 populations in Mexico reported a 12% population‐level extinction rate between 1975 and 2009 (Sinervo et al. 2010). The authors suggested these local extinctions may be the result of altered thermal niches, whereby lizards were unable to forage adequately to permit viable growth and reproduction (Sinervo et al. 2010).

To begin understanding the potential effects of climate change on snake and lizard species in western North America, we first projected the current climate niche of each species and then assessed how the locations of those niches may change in the future. We used species occurrence points and climate variables to model the geographic distribution of each species' fundamental or potential climate‐niche space under current or recent climate conditions and projected these relationships using past and future climate scenarios. Under each scenario, we generated maps depicting overall species richness and richness change (i.e., on the basis of climate suitability) between climate scenarios. We evaluated each species for future range changes and summarized information at the range‐wide level and for each of 47 state or provincial jurisdictions in western North America from Canada to Mexico. Given existing models and known reptile climate sensitivities, we hypothesized that: (1) species climate‐niche space would exhibit patterns of northern expansion and southern contraction in future scenarios, and (2) species would be more likely to occupy higher elevations in the future owing to warming temperatures and changing precipitation regimes (Cunningham et al. 2016).

## 2 Methods

### 2.1 Reptile Occurrence Points

We acquired snake and lizard occurrence points from open‐source data when available and from government‐owned data sets accessible through data‐sharing agreements (Appendix 1). Data were formatted and combined using ArcGIS software (ESRI, Redlands, CA, USA). We used Crother (2017) for species nomenclature, and we grouped sub‐species because of the potential for unreliable identification across time and observers. We filtered the dataset to include only data from the years 1981 to 2018. We removed redundant data by location (i.e., 1‐km climate grid cell) and species (i.e., identical species records from different data sources or multiple captures of the same species from a specific grid cell). We flagged and discarded records within the United States (U.S.) that fell ≥ 200 km from their documented Gap Analysis Project (GAP) range polygons (U.S. Geological Survey 2013b, 2018b), when available. We also discarded records that fell within 300 m of a state or county centroid (i.e., geographic center of a polygon) because examination found that the centroid coordinates had been used as an approximate location by various data sources for records without specific location information. We discarded records that did not have a year attribute and those with uncertain location or quality.

Candidate reptile species were selected for modeling if we had > 50 occurrence points (133 of 195 species: 68.2%; Appendix 2). Data‐deficient species that were not included in analyses were primarily found in the southern and on the eastern edge of our study extent (e.g., Mexico and southeastern U.S.). We combined four *Hypsiglena* species into one species group (i.e., genus) because of taxonomic uncertainty of historical records (Crother 2017), which resulted in 129 species and 1 genus for modeling (Appendix 2). Hereafter, we refer to these as 130 taxa as “species” for convenience.

### 2.2 Climate Data

We used the ClimateNA v5.5 tool to generate climate surfaces from a Digital Elevation Model (DEM) raster (U.S. Geological Survey and ESRI 2005; Wang et al. 2016). For each 1‐km grid cell across western North America, we generated average monthly temperature and precipitation values, resulting in 24 variables (Appendix 3). The climate variables were generated for four periods: past (1901–1930), recent (1981–2010), future mid‐century (2040–2069), and future late‐century (2070–2100). Future climate was derived using a 15‐model ensemble approach under two future emission scenarios (RCP 4.5 and RCP 8.5; Wang et al. 2016). Thus, six time‐by‐climate scenarios were examined for each species (one past, one recent, and four future). The tabular output data from ClimateNA were converted into rasters for subsequent climate niche modeling procedures.

### 2.3 Climate Niche Modeling

We performed the following analyses in R 3.4.3–3.5.1 and RStudio 1.1.383 (RStudio Team 2016; R Core Team 2017). We used the R package “biomod2 3.3‐7” for species modeling (Thuiller et al. 2009, 2016; Guisan, Thuiller, and Zimmermann 2017). Because of the size of the datasets and computations required for this analysis, we processed all models with the Yeti computer cluster (i.e., “supercomputer”) hosted by the USGS Core Science Systems Advanced Research Computing center (https://www.usgs.gov/advanced‐research‐computing). We chose to exclude geographic (e.g., latitude), topographic (e.g., elevation, slope, aspect), and biophysical (e.g., soils, rock outcrops, vegetation cover types) variables in our models for the following reasons: (1) temperature and precipitation covary with many of these variables (Bonan 2002; Daly et al. 2008; Harrison, Spasojevic, and Li 2020); (2) not all variables will change under future climate scenarios and those that do are mostly not currently available across our study area (Rigge et al. 2023); (3) the intent of this research was to assess species climate niches and predict future climate suitability, not to develop the best‐fitting species distribution models; and (4) we required datasets of commonly derived attributes that were available from Mexico to Canada. We ran 32 single models and one ensemble model for each species, resulting in 4290 total model runs.

We generated two replicates of pseudo‐absences using 50,000 random points for each species from our presence‐only dataset. For model evaluation, we withheld 30% of the occurrence points for each species' model run. This process was conducted twice for each of the two pseudo‐absence generations, resulting in four runs for each analytical technique. We modeled the climate niche for each species using eight analytical techniques, including artificial neural networks (ANN), classification tree analysis (CTA), functional data analysis (FDA), generalized additive model (GAM), generalized linear model (GLM), multivariate adaptive regression splines (MARS), random forest (RF), and surface range envelope (SRE). We evaluated the single models based on their true skill statistic (TSS). To reduce model‐based bias, we created a single ensemble model for each species by scenario combination using a weighted means approach. We included models in the aggregating procedure when they had TSS > 0.7 (Guisan, Thuiller, and Zimmermann 2017). Ensemble model performance was evaluated by the TSS score, specificity, and sensitivity. Variable importance values for the 24 climate variables were extracted for each species from the ensemble model.

### 2.4 Climate‐Niche Space Projections

We generated binary projections for all six climate scenarios with the ensemble models using the default methodology within “biomod2,” which selects a threshold that will maximize specificity and sensitivity. When available, we correlated our recent (1981–2010) climate‐niche space projections with published range and distribution information (U.S. Geological Survey 2013a, 2013b, 2018a, 2018b) to identify any modeling anomalies. We evaluated species' range changes by comparing the binary projection of suitable climate‐niche space for each of the six climate scenarios. We used the *biomod_rangesize* function to create a summary table of projection comparisons for each species.

### 2.5 Changes in Species' Climate‐Niche Space

For each species and scenario comparison, we identified whether its climate‐niche space: (1) had a net increase of at least 10%; (2) had a net decrease of at least 10%; or (3) remained stable with a change between −10% and 10%. We then compared the recent (1981–2010) climate‐niche space to the late‐century future RCP 8.5 (2070–2100) scenario to evaluate if a species' suitable climate‐niche space was projected to expand (little loss of suitable locations, with gains elsewhere), contract (loss of suitable locations, without gains elsewhere), remain stable (little change in suitable locations), or shift (expand or contract, while losing suitable locations). The predicted climate‐niche space centroid for each scenario was calculated using the R package “sdmtools 1.1‐221” and *cogravity* function (VanDerWal et al. 2014). We assessed range shifts between recent and future scenarios by evaluating the percent of pixels occupied in the recent scenario that were no longer predicted to be occupied in the future scenario in conjunction with the angle and distance between centroids of scenarios.

We then generated potential species richness rasters by stacking and summing the binary projections of climate‐niche space for each species. These maps represent the cumulative climate suitability locations of all modeled reptiles in our dataset and not true estimated species richness. However, for ease of communication we refer to the maps as potential richness maps and we generated one for each of the six climate scenarios to visualize changes through time and under different climate‐change outcomes. To evaluate richness change, we subtracted richness rasters between time steps. We identified “hotspots” and their changes visually.

### 2.6 Species Management Within and Across Jurisdictional Boundaries

We summarized which species were predicted to occur within each of 47 U.S. and Mexico states and districts and Canadian provinces (hereafter, “states”) per climate scenario. Presence was defined as having > 50 pixels of predicted suitable climate‐niche space within a state. For U.S. states, we compared our model‐derived, predicted state occurrence to those reported by Partners in Amphibian and Reptile Conservation (PARC; Pilliod and Wind 2008, Jones, Halama, and Lovich 2016), which are derived from Nature Serve (natureserve.org), regional herpetological expert opinion, and state biologist consultation. Changes in suitable climate area and location between scenarios were calculated as previously described. We then quantified elevation shifts within state boundaries by comparing elevation distributions within the predicted suitable locations between scenarios. Specifically, we compared the 25th and 75th percentile values to establish significance (Tukey 1977). For simplicity, in this paper, we focus on comparisons between the recent (1981–2010) and late‐century future RCP 8.5 (2070–2100) scenarios, but comparisons between all scenarios are available for download or interactive visualization (Jeffries et al. 2024a, 2024b).

## 3 Results

### 3.1 Climate Niche Modeling and Projections

**FIGURE 1:**
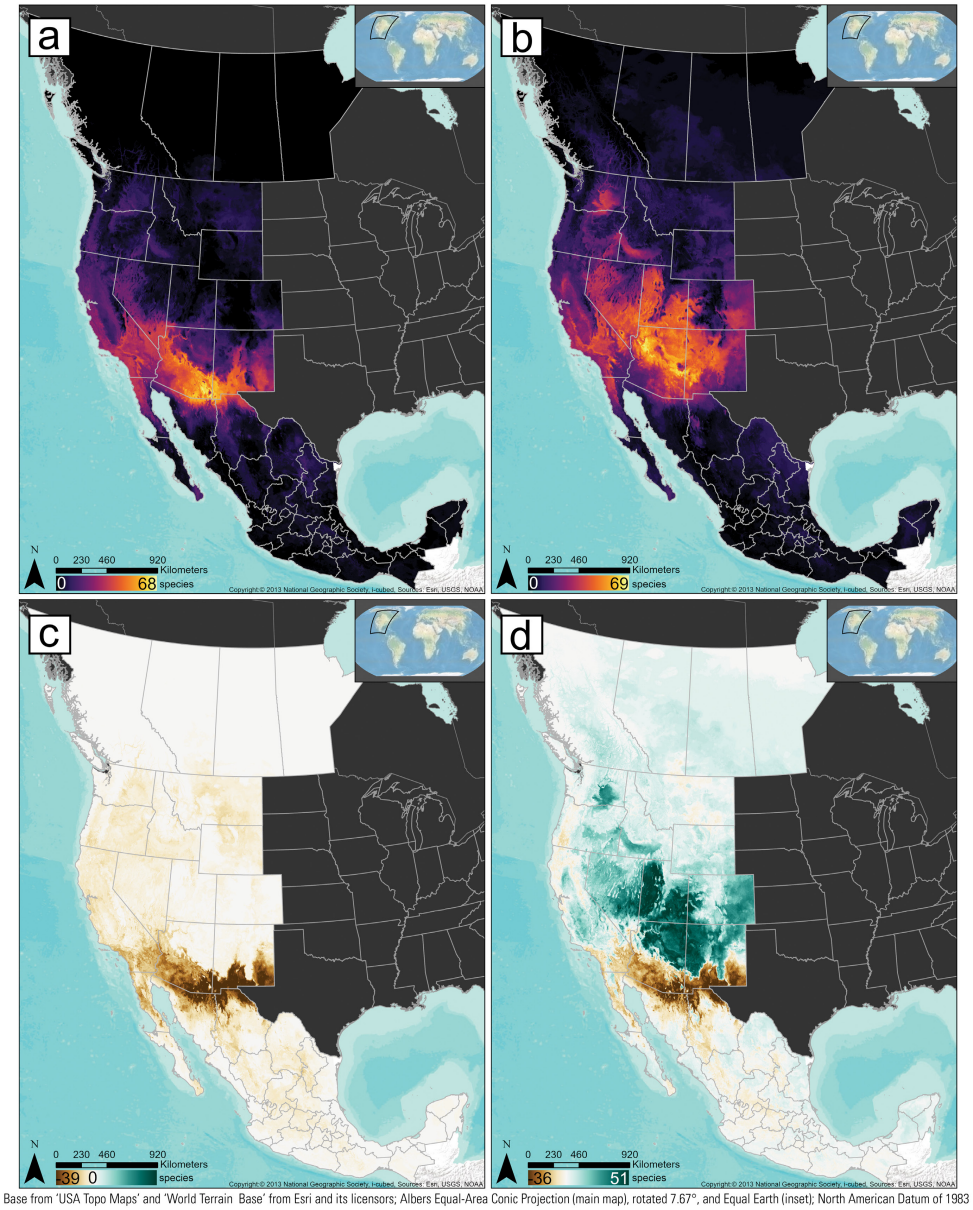
Binary climate‐niche rasters were combined across all species to create overall species richness and its geographic change between climate scenarios. Climate‐niche species‐richness map for (a) the recent climate scenario (1981–2010) and (b) late‐century future RCP 8.5 climate scenario (2070–2100). Climate‐niche species‐richness change between the late‐century future RCP 8.5 climate scenario (2070–2100) and the recent scenario (1981–2010) assuming (c) no dispersal ability for any species into novel areas, versus (d) full dispersal ability for any species into novel areas.

All 130 species yielded at least three single models with a TSS > 0.7; thus we created ensemble models for all 130 species for six climate scenarios: past (1901–1930), recent (1981–2010), mid‐century future RCP 4.5 (2040–2069), mid‐century future RCP 8.5 (2040–2069), late‐century future RCP 4.5 (2070–2100), and late‐century future RCP 8.5 (2070–2100) (Figure 1a,b; model performance summaries: Appendices 4 and 5; Jeffries et al. 2024a, 2024b). The top three climate variables across all models tended to include January average temperature (average variable importance ±SD: 0.21 ± 0.14), June average temperature (average variable importance ±SD: 0.20 ± 0.21), and June average precipitation (average variable importance ±SD: 0.19 ± 0.18; Appendix 6; Jeffries et al. 2024a).

### 3.2 Changes in Species' Climate‐Niche Space

Summaries of suitable climate area and change in location between scenarios are available for each species in Jeffries et al. (2024a, 2024b, https://geonarrative.usgs.gov/reptileclimateniche). Relative to the recent (1981–2010) climate‐niche projections, 68% of species are predicted to gain climate‐niche space regardless of time or climate scenario. On average, by late‐century (2070–2100), these species are predicted to gain an additional 70% (range: 20%–732%) of suitable climate space relative to their recent climate niche under the RCP 4.5 scenario and 116% (range: 10.5%–1513%) under the RCP 8.5 scenario. Conversely, only 8.5% of species are projected to lose climate‐niche space in all future climate scenarios. On average, by late century, these species are predicted to lose 41% (range: 13%–100%) of their recent climate‐niche space under the RCP 4.5 scenario and 65% (range: 17%–100%) under the RCP 8.5 scenario. Only six species (4.6%) are projected to maintain stable climate‐niche space under all future emission scenarios.

**FIGURE 2:**
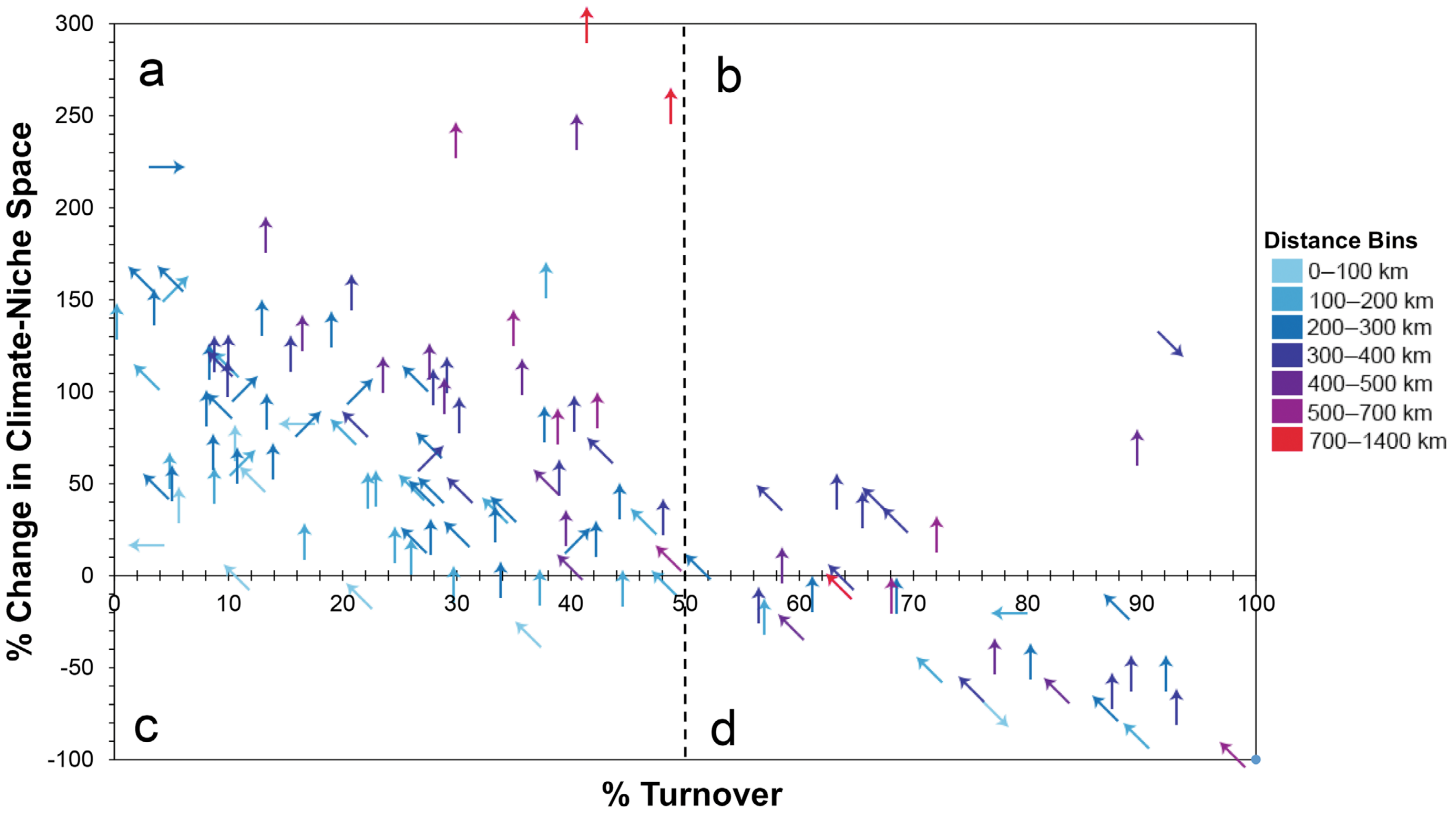
Species that have a projected increase in suitable climate area and (a) maintain most of the original pixels, versus (b) lose over half of the original pixels. Species that have a projected decrease in suitable climate area and (c) maintain most of their original pixels, versus (d) lose most of their original pixels. Percent change of the climate‐niche size for each species between the recent (1981–2010) and late‐century future RCP 8.5 climate scenario (2070–2100) is on the *y*‐axis and the percent of the pixels from the recent scenario that are no longer predicted as viable in the future scenario is on the *x*‐axis. Arrows indicate general direction of the centroid shift between the recent and future scenario (up is north, down is south, left is west, and right is east). Arrow color represents the distance of the centroid shift. The dashed line at 50% turnover was added to highlight groups of species. An interactive version of this figure is available (Jeffries et al. 2024a).

**FIGURE 3:**
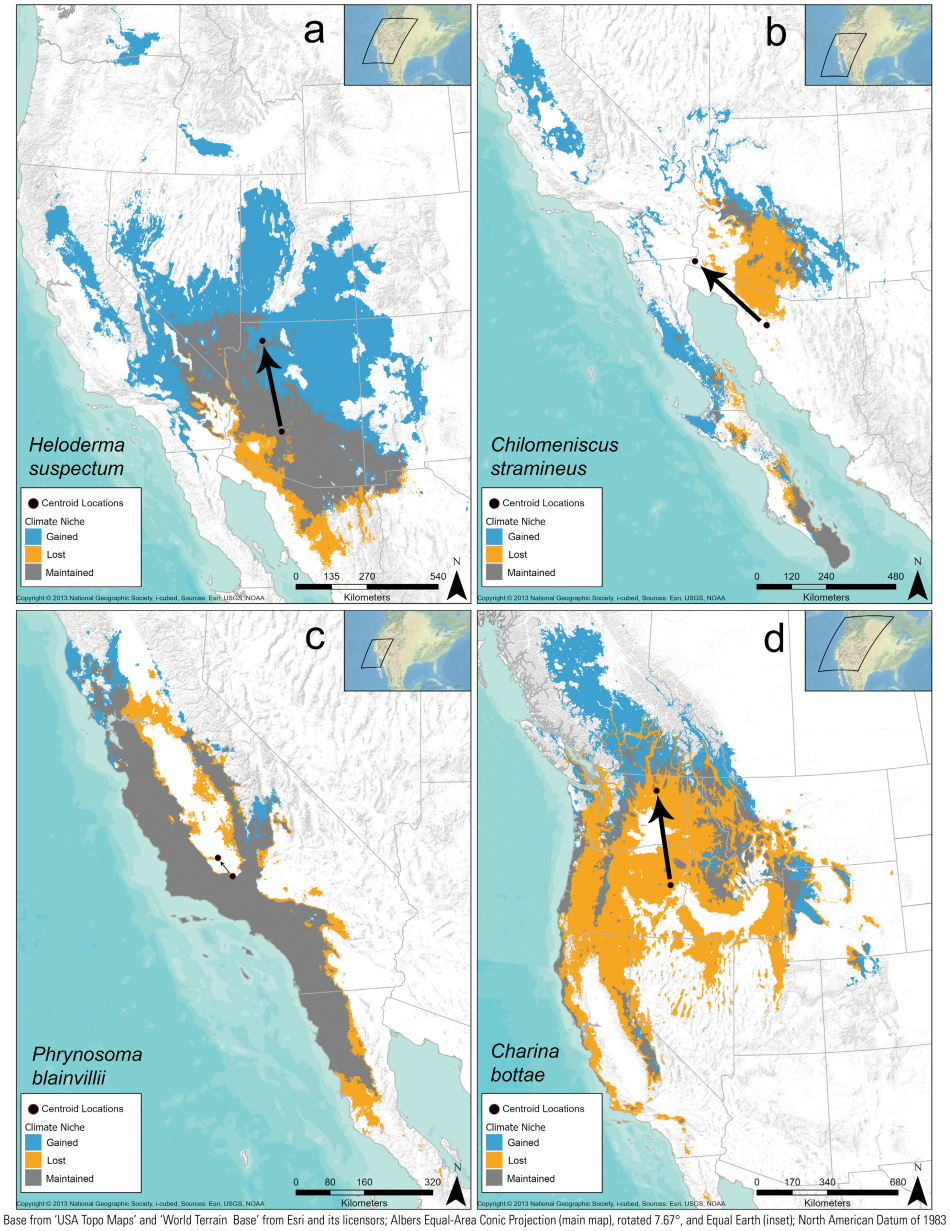
Four species exemplifying the climate‐niche space that is maintained, gained, or lost when comparing the recent (1981–2010) to the late‐century future RCP 8.5 climate scenario (2070–2100). (a) Gila monster (*Heloderma suspectum*) is an example of an expanding species. (b) Variable sandsnake (*Chilomeniscus stramineus*) represents a shifting species. (c) Blainville's horned lizard (*Phrynosoma blainvilli*) is a generally stable species. (d) Northern rubber boa (*Charina bottae*) is a contracting species. These illustrations are available for all modeled species (Jeffries et al. 2024b).

When we accounted for whether recently suitable climate space was maintained through time and climate scenario, we found that under the late‐century future RCP 8.5 scenario, most species (85/130) are predicted to expand their climate space by at least 10%, while maintaining at least 50% of their original niche space (e.g., Figure 2a). In contrast, nine species are predicted to increase suitable space by at least 10%, but also to lose at least 50% of their recently suitable area (i.e., shift; Figure 2b). Twenty‐one species are predicted to decrease suitable space by at least 10% while losing at least 50% of their original niche space (i.e., contract; Figure 2d, Table 1). Two additional species are projected to have range reductions of at least 10% overall, but also to lose less than 50% of their recently suitable area (i.e., contract; Figure 2c). Five species had stable climate‐niche area sizes, but shifting centroid locations (i.e., Figure 2d near the *x*‐axis). The remaining eight species had relatively stable climate‐niche sizes and centroid locations (i.e., Figure 2b near the *x*‐axis). The centroid values of suitable climate distributions largely shifted northward an average of 313 ± 18 km. To visualize these patterns, we show examples using the Gila Monster (*Heloderma suspectum*) as a species that is predicted to have an expanding climate‐niche space (Figure 3a), the Variable Sandsnake (*Chilomeniscus stramineus*) as a shifting species (Figure 3b), the Blainville's Horned Lizard (*Phrynosoma blainvillii*) as a stable species (Figure 3c), and the Northern Rubber Boa (*Charina bottae*) as a species that is predicted to lose suitable climate area (Figure 3d).

**TABLE 1:** Twenty‐one species projected to lose at least 10% of their suitable climate‐niche space and lose most (> 50%) of their original range between the recent (1981–2010) and late‐century future climate RCP 8.5 climate scenario (2070–2100).

Scientific name	Range size					Percent change in future scenario		Range change
	Current	Future	Loss	Gain	Maintain	Lost	Gained	
Common name								
*Sceloporus arenicolus* Dunes Sagebrush Lizard	4322	0	4322	0	0	100.00	0.00	−100.00
*Phrynosoma douglasii* Pygmy Short‐horned Lizard	188,650	4897	184,792	1039	3858	97.96	0.55	−97.40
*Anniella pulchra* Northern Legless Lizard	28,923	3722	25,896	695	3027	89.53	2.40	−87.13
*Thamnophis couchii* Sierra Gartersnake	76,300	18,958	58,877	1535	17,423	77.17	2.01	−75.15
*Aspidoscelis flagellicauda* Gila Spotted Whiptail	36,205	10,057	31,429	5281	4776	86.81	14.59	−72.22
*Sceloporus virgatus* Striped Plateau Lizard	22,963	6606	21,362	5005	1601	93.03	21.80	−71.23
*Crotalus pricei* Twin‐spotted Rattlesnake	46,652	17,456	38,514	9318	8138	82.56	19.97	−62.58
*Crotalus willardi* Ridge‐nosed Rattlesnake	21,943	8226	19,176	5459	2767	87.39	24.88	−62.51
*Sceloporus jarrovii* Spiny Lizard	178,006	68,042	133,645	23,681	44,361	75.08	13.30	−61.78
*Sceloporus slevini* Slevin's Bunchgrass Lizard	25,788	12,126	22,968	9306	2820	89.07	36.09	−52.98
*Aspidoscelis uniparens* Desert Grassland Whiptail	70,471	33,148	64,910	27,587	5561	92.11	39.15	−52.96
*Crotalus lepidus* Rock Rattlesnake	130,401	63,573	93,115	26,287	37,286	71.41	20.16	−51.25
*Sceloporus graciosus* Common Sagebrush Lizard	458,409	245,176	367,842	154,609	90,567	80.24	33.73	−46.52
*Charina bottae* Northern Rubber Boa	440,424	248,029	339,587	147,192	100,837	77.11	33.42	−43.68
*Crotalus molossus* Western Black‐tailed Rattlesnake	321,340	230,966	190,524	100,150	130,816	59.29	31.17	−28.12
*Aspidoscelis sonorae* Sonoran Spotted Whiptail	47,145	36,709	26,835	16,399	20,310	56.92	34.78	−22.14
*Senticolis triaspis* Green Ratsnake	86,733	69,096	67,975	50,338	18,758	78.37	58.04	−20.34
*Phrynosoma mcallii* Flat‐tailed Horned Lizard	15,033	12,475	13,200	10,642	1833	87.81	70.79	−17.02
*Elgaria kingii* Madrean Alligator Lizard	115,286	96,979	65,054	46,747	50,232	56.43	40.55	−15.88
*Lampropeltis splendida* Desert Kingsnake	119,582	106,743	81,925	69,086	37,657	68.51	57.77	−10.74
*Coluber constrictor* North American Racer	454,753	406,524	309,533	261,304	145,220	68.07	57.46	−10.61

*Note:* Species are ordered by range change (%). Size values represent the count of pixels with a 1‐km grid size. Current range sizes include the pixels predicted to be occupied in the recent (1981–2010) scenario, whereas the future sizes are those predicted to be occupied in the late‐century future RCP 8.5 scenario. Loss is the number of pixels no longer predicted to be occupied in the future scenario. Gain represents pixels not predicted to be occupied in the first scenario but are occupied in the future scenario. Maintain pixels are predicted to be occupied in both scenarios. Percent lost is the percent of currently occupied pixels that are lost (Loss/(Loss + Maintain)). The percent gained is the percent of new pixels in relation to the current range size (Gain/(Loss + Maintain)). Species Range Change is calculated by subtracting the percent gained from the percent lost. These data are available for all species and scenario comparisons and within the interactive graphical supplement (Jeffries et al. 2024a, 2024b).

In the recent projection of richness, the highest values were found in southern Arizona and northern Mexico (Figure 1). By mid‐century, under the RCP 8.5 emission scenario, richness shifts northward, with increases in northern Arizona and northward. The trend continued in the late‐century future scenario, with richness losses being most evident in southern Arizona and northern Mexico, as species richness expands and shifts northward. The largest increases in species richness are projected in northeastern Arizona, southeastern Utah, western Utah, northwestern New Mexico, and southwestern Colorado. In contrast, fewer new climate‐niche spaces expand into Canadian provinces (Figure 1).

### 3.3 Species Management Within and Across Jurisdictional Boundaries

**FIGURE 4:**
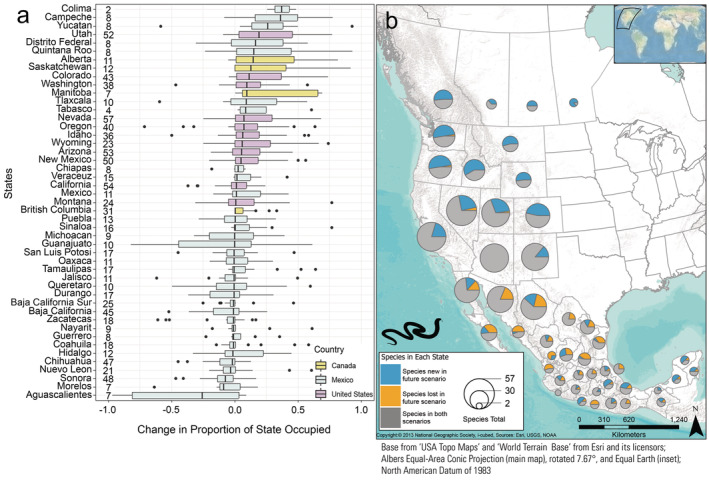
State‐level summaries of climate‐niche changes of snake species between recent (1981–2010) and late‐century future RCP 8.5 climate scenarios (2070–2100). (a) Change in proportion of each state where snake species are predicted to occur between the two time periods. A positive value for an individual species represents an increase in the proportion of the state the species is predicted to occupy from the recent‐to‐future scenario, with the 25th and 75th percentiles, median line, whiskers extending to 1.5‐times the interquartile range, and outliers displayed as points. Sample size (no. species) for each state is displayed to the right of the name. (b) Counts of snake species predicted to be gained, lost, or maintained in the future scenario per state. The size of the pie chart represents the number of species for each jurisdiction. An interactive version of this figure is available (Jeffries et al. 2024a).

**FIGURE 5:**
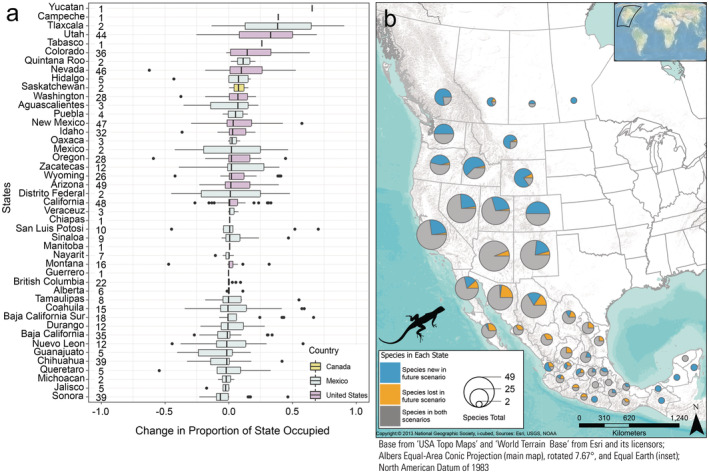
State‐level summaries of climate‐niche changes of lizard species between recent (1981–2010) and late‐century future RCP 8.5 climate scenarios (2070–2100). (a) Change in proportion of each state where lizard species are predicted to occur between the two time periods. A positive value for an individual species represents an increase in the proportion of the state the species is predicted to occupy from the recent‐to‐future scenario, with the 25th and 75th percentiles, median line, whiskers extending to 1.5‐times the interquartile range, and outliers displayed as points. Sample size (no. species) for each state is displayed to the right of the name. (b) Counts of lizard species predicted to be gained, lost, or maintained in the future scenario per state. The size of the pie chart represents the number of species for each jurisdiction. An interactive version of this figure is available (Jeffries et al. 2024a).

**FIGURE 6:**
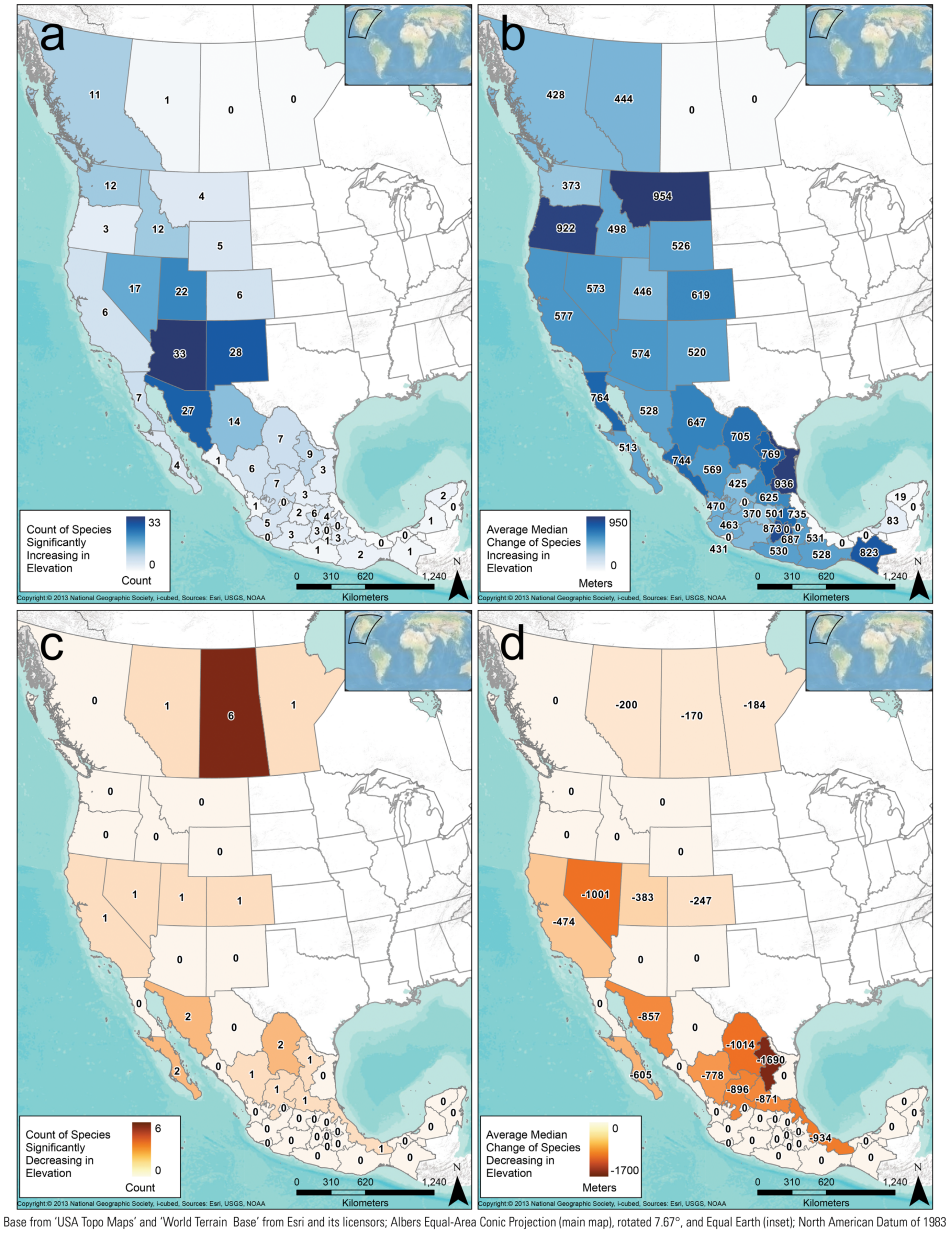
Elevational (m) distributions for all squamate species climate niche compared between the recent (1981–2010) and late‐century future RCP 8.5 climate scenarios (2070–2100) within each state. The 25th and 75th percentiles were compared to evaluate significance. Scenarios that do not overlap between the 25th and 75th percentiles were considered significantly different. (a) The count of species in each state where the distribution of elevation significantly increased. (b) The average median change between scenarios for each species that increased in elevation. (c) The count of species in each state where the distribution of elevation significantly decreased. (d) The average median change between scenarios for each species that decreased in elevation. An interactive version of this figure is available (Jeffries et al. 2024a).

Species occurrence patterns within geographically defined jurisdictions were complex, thus here we report comparisons only between the recent (1981–2010) and late‐century future RCP 8.5 (2070–2100) scenarios. Modeled species occurrences for the recent scenario were correctly predicted in a state an average of 83.1% of the time. When species were predicted to occur in the recent scenario, they were also predicted to occur (based on climate suitability) in that state in the future scenario 86% of the time (Figures 4b and 5b), and we designated those species as having a stable occurrence in the state (e.g., gray portions in Figures 4b and 5b). Those “stable‐occurrence species” lost an average of 38 ± 1.08% (median: 28.7, range: 0–100) of their originally occupied climate‐niche space but had an average overall change of 1231 ± 173% (median: 34%, range: −99 to 72,053) of suitable area in those states. In addition, 26% of the stable‐occurrence species/state combinations are predicted to significantly increase in elevation within the state. In contrast, the majority (72%) are predicted to occupy significantly similar elevations, and only 2% were predicted have suitable climate ranges that decreased in elevation (Figure 6).

The 14% of species‐by‐state combinations where the species was predicted to occur recently and subsequently predicted to have no suitable climate in future scenarios tended to have small areas of suitable climate recently (i.e., fewer recent occurrence pixels: mean: 2594 ± 534, median: 523, range: 54–72,678) than those species predicted to persist (mean: 20,066 ± 800, median: 9194, range: 51–137,774). This value (14%) represents 176 of 1251 species predicted to occur in a state (note that 1251 is greater than the total number of distinct species modeled [i.e., 130] because most species occur in more than one state). Most species in Baja California Sur, Mexico, are expected to lose suitable climate area in the state during the future scenario, and half of those species had very small areas of suitable climate (< 500‐occurrence pixels or about 1.8% of the state) recently. Some currently widespread species are also predicted to lose all suitable climate‐niche space in a few states. For example, the Pygmy Short‐horned Lizard (*Phrynosoma douglasii*) is projected to lose its entire climate‐niche space in Alberta, Canada, and the U.S. states of California, Idaho, Montana, Nevada, Oregon, Utah, and Wyoming, while retaining only a small portion in Washington, USA and British Columbia, Canada. The North American Racer (*Coluber constrictor*) is projected to lose its entire climate‐niche space in Baja California, Mexico, Nevada, and Utah, but maintain suitable areas in 10 other states. The Dunes Sagebrush Lizard (*Sceloporus arenicolus*) was predicted to lose all suitable climate‐niche space across the entire study area, though its persistence in Texas, to the east of the study area, was not modeled (U.S. Geological Survey 2018a, 2018b; Jeffries et al. 2024a). This species was recently listed as Endangered by the U.S. Fish and Wildlife Service (Federal Register 2024).

Overall, 40 states (85%) are predicted to lose at least one species under the future climate scenario. Sonora and Chihuahua, Mexico, are predicted to lose suitable areas for the largest numbers of species (18 and 16, respectively). Conversely, nearly all states (89%) are predicted to gain suitable areas for additional species in the future. In the U.S., Idaho and Colorado are predicted to gain suitability for the most species, 40 and 38, respectively.

## 4 Discussion

Lizards and snakes are an understudied group of tetrapods in North America, which creates a challenge for natural‐resource managers tasked with managing species, habitats, and planning mitigation actions in response to myriad threats including changing climate. By taking a taxonomically comprehensive approach to assessing recent and potential future reptile niches in western North America, we aimed to provide useful information to inform natural‐resource managers across 47 geographically defined jurisdictions that have responsibility for species‐conservation and habitat‐management decisions. Our findings are congruent with several other studies suggesting a northward or upward climate‐based shift for many species (Araújo, Thuiller, and Pearson 2006; Barrows 2011; Morena‐Rueda et al. 2012). Furthermore, in western North America, our results suggest potentially widespread changes in squamate species distributions with species‐scale losses and gains at state and provincial scales. With an uncertain capacity for individual lizard and snake species to persist‐in‐place or shift‐in‐space in response to climate change (Thurman et al. 2022), our results may help inform natural resource agencies within specific jurisdictional boundaries to prepare for new species arriving, current species extirpation, possibly assisted migration, and shifts in species distributions within jurisdictions, including into areas at higher elevations.

We identified several species with a shifted or contracted climate‐niche space within the next 50 years, which could create new species‐conservation challenges (Table 1). Interestingly, some of these species have not been previously identified as particularly rare and climate sensitive, thus they have not drawn management attention in prior assessments using different analysis approaches and spatial scales (e.g., *Phrynosoma douglassi*, *Sceloporus graciosus*, *Charina bottae*, *Coluber constrictor*: Mims et al. 2018). Hence, the current analyses identify potentially underappreciated broad‐scale effects of climate change. Although range shifts and distributional changes are common over the evolutionary history of most species (Kafash et al. 2020), many species exist in isolated habitat “islands” (Jones, Kepner, and Martin 1985). Furthermore, the modern world has new barriers to dispersal due to anthropogenic activities that reduce landscape connectivity (Tan, Herrel, and Rödder 2023), and thus species that are predicted to shift‐in‐space from their current range may not be able to disperse into new areas of suitable climate because of anthropogenic barriers (Driscoll 2004; Lasky, Jetz, and Keitt 2011; Kay et al. 2016; Brehme, Hathaway, and Fisher 2018; Paterson et al. 2019). Even if species can disperse, they may not be able to find climate refugia or meet basic physiological requirements when dispersing through suboptimal habitat conditions (Hillman et al. 2014; Boyle et al. 2016). Despite dispersal limitations, some squamate species have been documented to shift their range with shifting climate regimes (Frishkoff et al. 2019). A meta‐analysis of several species found shifts occurring at the rate of 6.1–19.9 km/decade (Parmesan and Yohe 2003; Chen et al. 2011). In addition to distance, elevation shifts have also been observed for some squamate species (Raxworthy et al. 2008; Chen et al. 2011). Where topographic variation provides climate refuge, climate niches may be available for some species in relatively close proximity but at higher elevations, as indicated by our analysis. This type of refuge has its realized limits and is dependent on concomitant shifts in critical resources (e.g., vegetation, prey) and availability of critical resources (e.g., hibernacula).

Across 130 squamate species, the most commonly important variables defining species' climate‐niche space in our analyses were January temperature, June temperature, and June precipitation. The importance of January temperature is consistent with prior research (Williams, Henry, and Sinclair 2015; Hatten et al. 2016), and changes in these January and June conditions could shift the timing of life‐history transitions, such as hibernation, breeding, and foraging, across seasons. Western North America has considerable seasonal and spatial climate variability. We might expect species found in areas with more heterogeneous climate conditions to be better suited to adjust to future changes, whereas species found in climate zones with more homogeneous conditions (e.g., equatorial) could be more sensitive to baseline changes. A recent meta‐analysis, however, found that climate‐vulnerable species occur in a variety of locations and from distinct phylogenies (Diele‐Viegas et al. 2020).

Inference and prediction using climate‐niche models can be influenced by the parameters selected and variables used (Zurell et al. 2020). There are several ways to characterize climate and seasonality, and we chose to use monthly climate data instead of mean annual temperature, annual temperature range, and total annual precipitation to identify possible mechanistic relationships with seasonal changes in animal life‐history functions and processes. Although we evaluated monthly climate data, we were unable to assess more nuanced effects of climate change, such as seasonality or the occurrence and duration of extreme weather events (e.g., droughts; Zani and Stein 2018; regional extreme temperature events, e.g., 2021 “heat dome”: U.S. Department of Agriculture 2021). These episodic events are difficult to predict spatially under future scenarios, except as probabilities, yet could have significant effects on biota.

The effects of shifting climates on colonization and extinction processes are complex and difficult to predict because of the plurality of organismal or population‐level factors that influence species' adaptive capacity, including but not limited to genetic capacity for adaptation, physiology, morphology, and phenotypic plasticity. Furthermore, many effects will be context dependent because of quality of habitats and site‐to‐microsite conditions such as thermal‐and‐moisture characteristics of microhabitats used for breeding, foraging, and overwintering, habitat conditions in dispersal areas, prey, and predators.

Daily temperature amplitude and nighttime temperatures have been shown to affect reptiles, primarily through embryonic development and metabolic pathways (Booth 2018; Rutschmann et al. 2024). For example, studies of Side‐blotched Lizards (*Uta stansburiana*), a widespread species in western North America, indicated that warmer temperatures, especially nighttime minimum temperatures, during egg development could increase reproductive output and thus, lizard fitness (Clarke and Zani 2012). However, warm‐and‐dry conditions, such as during extended droughts, can reduce body size and condition in females and lead to reproductive failure and no population recruitment (Zani and Stein 2018). This effect was not associated with a lack of prey but rather a lack of surface water for drinking. Autumn growing‐season length can increase body size in side‐blotched lizards; larger lizards are more likely to survive winter (because of their higher energy storage prior to dormancy) and larger females are more likely to reproduce successfully (Zani 2005, 2008). Finally, warmer winter temperatures can reduce survival, apparently by depleting glycogen levels in the liver (Zani et al. 2012). These results demonstrate the ecological complexity of changing environmental conditions associated with climate and indicate that for side‐blotched lizard species or populations at the northern limits of their ranges, certain conditions, such as warmer nighttime summer temperatures and longer growing seasons, may offset negative effects of drought or warmer winters by altering the timing of reproduction, reproductive output, growth, and recruitment (Zani and Rollyson 2011). This illustrates how the long‐term persistence of some populations may depend on how specific aspects of a changing climate affect certain life stages or timing in an animal's life history (Thurman et al. 2020). These effects can be further confounded by local adaptations and animal traits (Smith, Zani, and French 2019).

Additional research would be needed to understand thermal tolerances, abiotic tolerances, and adaptive capacities of most squamate species across our study extent because the temporal and spatial scales of our analyses are not sufficient to describe proximate mechanisms as the interactions between climate change and life‐history functions. Nevertheless, the broadscale climate‐related range changes we project could be used to generate downscaled hypotheses that could be tested at spatial scales relevant for individual species. Such empirical studies could provide important contexts for natural resource planning of species and habitats at potential risk. For example, for each management jurisdiction, managers could use study results to identify areas of overlapping present and future species‐specific climate‐niche space and prioritize areas for targeted conservation activities (Jeffries et al. 2024a). Wildlife managers could also use study results to identify hotspots of climate suitability for multiple species and areas that provide climate refugia (i.e., climate stable, or areas suitable now and in the future) and may be particularly important for species persistence (Morelli et al. 2016). Interstate to international scale planning may be needed for species with large ranges or those predicted to shift long distances. As management actions that cross jurisdictions become necessary or common, larger‐scale “top‐down” planning can also be informed by our results (e.g., Popescu et al. 2013). Ultimately, our results can be used to assess landscape connectivity between climate scenarios and inform development of conservation priorities that sustain squamate species experiencing climate‐driven range shifts (Littlefield et al. 2019; Zhu et al. 2021).

## Author Contributions

**David S. Pilliod:** conceptualization (lead), funding acquisition (lead), project administration (lead), resources (lead), supervision (lead), writing – original draft (equal), writing – review and editing (equal). **Michelle I. Jeffries:** conceptualization (equal), data curation (lead), formal analysis (lead), investigation (lead), methodology (lead), project administration (supporting), software (lead), validation (lead), visualization (lead), writing – original draft (equal), writing – review and editing (equal). **Robert S. Arkle:** conceptualization (supporting), formal analysis (supporting), methodology (supporting), writing – review and editing (equal). **Deanna H. Olson:** conceptualization (supporting), data curation (supporting), investigation (supporting), writing – review and editing (equal).

## Conflicts of Interest

The authors declare no conflicts of interest.

### Open Research Badges

This article has earned an Open Data badge for making publicly available the digitally‐shareable data necessary to reproduce the reported results. The data is available at https://doi.org/10.5066/P9ZGRGE4.

## Data Availability

The species occurrence data used in the analysis for this study were collected with appropriate data‐sharing agreements and from sources openly available to the public. See Appendix 1 for a list of data sources. Climate data were derived using the Climate North America tool (ClimateNA v5.5; http://climatena.ca/) and a Digital Elevation Model raster (https://www.sciencebase.gov/catalog/item/50db7222e4b061270600b8a4). Summary data and model outputs can be found at https://doi.org/10.5066/P9ZGRGE4. The interactive graphical supplement is available at https://geonarrative.usgs.gov/reptileclimateniche. Full citations can be found in the References.

## Appendix 1

A list of data sources and contributors. Data sources were publicly available (HerpNet2 2012; GBIF 2016; BISON 2017), except for those with an asterisk, which were available upon approval or a data‐sharing agreement with the data owners.

**Table:**

Data sources and contributors
A. Linder*
A. Safranek*
A. St. John*
Alberta Fish and Wildfire Service*
American Museum of Natural History*
Arizona Game and Fish Department*
B. Bean*
B. Budd*
B. Ralston*
B. Schneider*
Berkeley Museum of Vertebrate Zoology*
Biodiversity Information Serving our Nation
British Columbia Ministry of Environment*
Bureau of Land Management*
C. & R. Grove*
C. Peterson*
California Academy of Sciences*
California Department of Fish and Wildlife*
Carnegie Museum*
D. Bilsland*
D. Fitcher*
D. Miller*
D. Quinney*
Douglas County Museum*
F. Uhler*
G. Beauvais*
G. Harold*
G. Pendlebury*
Global Biodiversity Information Facility
H. Fitch*
Hayden‐Wing*
HerpNet2 (now VertNet)
Idaho Citizen Science Data*
Idaho Department of Fish and Game*
Idaho Fish and Wildfire Service*
Idaho Fish and Wildlife Information Systems*
Idaho Herp Database*
Idaho Natural Heritage Program*
Idaho Power Company*
Idaho State University*
iNaturalist
J. Beck*
J. Cossel*
J. Engle*
J. Hawk*
J. Munger*
J. Weaver*
J. Yeo*
Kanisku National Forest*
Kansas University Natural History Museum*
L. Diller*
L. Murphy*
L. Powers*
Los Angeles County Museum of Natural History*
M. Mathis*
Montana Natural History Database*
National Parks Service*
Natural Heritage New Mexico*
Natural Resource Information System*
Nevada Department of Wildlife*
Orchard Combat Training Center*
Oregon Biodiversity Information Center*
Oregon Bureau of Land Management*
Oregon Department of Fish and Wildfire*
Oregon State University*
P.M. de Laubenfels*
Pacificorps*
Portland State University*
R. & D. Brokken*
R. Clark*
R. Davis*
R. Hoyer*
R. Jones*
R. Kiester*
R. Mason*
R. Nussbaum*
Saskatchewan Ministry of Environment*
Smithsonian Institution's National Museum of Natural History*
Southern Oregon University*
U.S. Geological Survey Gap Analysis Project*
University of Michigan*
University of Puget Sound*
University of Texas at Arlington*
Utah Natural Heritage Program*
Utah State University*
W. Haas*
W. Turner*
Washington Department of Fish and Wildlife*
Washington Department of Natural Resources*
Wyoming Cooperative Fishery and Wildlife Research Unit*
Wyoming Department of Fish and Game*
Wyoming Natural Diversity Database*

## Appendix 2

The list of species that occurrence point‐location data was collected for (Appendix 1). The “Count of All Species Points” column totals the number of points we collected, regardless of quality. The “Points with No Quality Flag, Any Date” subsets to those points without a known or obvious data quality issue. The “Points with No Quality Flag, 1981–2010” subsets the data to those from the years from 1981 to 2010. Lastly, the “Points with No Quality Flag, 1981–2010, One per Climate Grid‐cell” further reduces list to include only one point per‐species in each 1‐km climate grid cell used for modeling. The final column indicates if the species was included for climate‐niche modeling and spatial projection.

**Table:**

Scientific name	Common name	Count of all species points	Points with no quality flag, any date	Points with no quality flag, 1981–2010	Points with no quality flag, 1981–2010, one per climate grid‐cell	Species niche modeled and projected?
*Anniella pulchra*	Northern Legless Lizard	1096	766	228	157	Yes
*Arizona elegans*	Glossy Snake	3610	2676	1078	861	Yes
*Aspidoscelis exsanguis*	Chihuahuan Spotted Whiptail	112	112	99	60	Yes
*Aspidoscelis flagellicauda*	Gila Spotted Whiptail	264	143	109	77	Yes
*Aspidoscelis gularis*	Common Spotted Whiptail	585	302	144	116	Yes
*Aspidoscelis hyperythra*	Orange‐throated Whiptail	1193	775	306	211	Yes
*Aspidoscelis inornata*	Little Striped Whiptail	614	400	214	143	Yes
*Aspidoscelis motaguae*	Giant Whiptail	1	1	0	0	No
*Aspidoscelis neomexicana*	New Mexico Whiptail	237	131	32	27	No
*Aspidoscelis neotesselata*	Colorado Checkered Whiptail	52	19	13	13	No
*Aspidoscelis scalaris*	Plateau Spotted Whiptail	98	92	1	1	No
*Aspidoscelis sexlineata*	Six‐lined Racerunner	291	62	34	33	No
*Aspidoscelis sonorae*	Sonoran Spotted Whiptail	874	678	498	267	Yes
*Aspidoscelis stictogramma*	Giant Spotted Whiptail	379	255	180	113	Yes
*Aspidoscelis tesselata*	Common Checkered Whiptail	410	162	59	52	Yes
*Aspidoscelis tigris*	Tiger Whiptail	10,111	8863	4952	2984	Yes
*Aspidoscelis uniparens*	Desert Grassland Whiptail	1030	816	606	328	Yes
*Aspidoscelis velox*	Plateau Striped Whiptail	684	430	244	197	Yes
*Aspidoscelis xanthonota*	Red‐backed Whiptail	39	36	28	23	No
*Bogertophis rosaliae*	Baja California Ratsnake	28	27	8	8	No
*Bogertophis subocularis*	Trans‐Pecos Ratsnake	377	90	30	24	No
*Callisaurus draconoides*	Zebra‐tailed Lizard	22,216	19,901	11,598	3185	Yes
*Charina bottae*	Northern Rubber Boa	2726	2199	1389	1192	Yes
*Charina umbratica*	Southern Rubber Boa	65	10	4	4	No
*Chilomeniscus stramineus*	Variable Sandsnake	592	467	216	113	Yes
*Chionactis occipitalis*	Mohave Shovel‐nosed Snake	2487	2154	996	534	Yes
*Chionactis palarostris*	Sonoran Shovel‐nosed Snake	127	99	30	15	No
*Cnemidophorus lemniscatus*	Rainbow Whiptail	1	1	0	0	No
*Coleonyx brevis*	Texas Banded Gecko	388	171	63	57	Yes
*Coleonyx switaki*	Switak's Banded Gecko	47	30	18	18	No
*Coleonyx variegatus*	Western Banded Gecko	4855	4339	2801	1441	Yes
*Coluber bilineatus*	Sonora Whipsnake	588	444	356	204	Yes
*Coluber constrictor*	North American Racer	5186	4527	3174	2313	Yes
*Coluber flagellum*	Coachwhip	3541	3129	1764	1362	Yes
*Coluber fuliginosus*	Baja California Coachwhip	228	207	43	40	No
*Coluber lateralis*	Striped Racer	1140	867	451	386	Yes
*Coluber taeniatus*	Striped Whipsnake	1887	1396	722	574	Yes
*Contia longicauda*	Forest Sharp‐tailed Snake	27	27	12	12	No
*Contia tenuis*	Common Sharp‐tailed Snake	978	818	473	392	Yes
*Cophosaurus texanus*	Greater Earless Lizard	2251	1566	611	397	Yes
*Crotalus adamanteus*	Eastern Diamond‐backed Rattlesnake	2	1	0	0	No
*Crotalus atrox*	Western Diamond‐backed Rattlesnake	5503	4311	3307	1580	Yes
*Crotalus cerastes*	Sidewinder	3504	3048	1608	1113	Yes
*Crotalus cerberus*	Arizona Black Rattlesnake	208	203	169	98	Yes
*Crotalus lepidus*	Rock Rattlesnake	1003	719	438	213	Yes
*Crotalus mitchellii*	Speckled Rattlesnake	1412	1152	558	444	Yes
*Crotalus molossus*	Western Black‐tailed Rattlesnake	2181	1733	1309	686	Yes
*Crotalus oreganus*	Western Rattlesnake	6983	6123	4211	2786	Yes
*Crotalus pricei*	Twin‐spotted Rattlesnake	566	483	321	86	Yes
*Crotalus ruber*	Red Diamond Rattlesnake	1332	1023	335	278	Yes
*Crotalus scutulatus*	Mohave Rattlesnake	3247	2601	1826	1092	Yes
*Crotalus* sp.	Rattlesnake	190	185	105	92	No
*Crotalus stephensi*	Panamint Rattlesnake	269	269	269	215	Yes
*Crotalus tigris*	Tiger Rattlesnake	1304	1209	1095	189	Yes
*Crotalus viridis*	Prairie Rattlesnake	9116	6308	4757	3038	Yes
*Crotalus willardi*	Ridge‐nosed Rattlesnake	309	251	151	75	Yes
*Crotaphytus bicinctores*	Great Basin Collared Lizard	21,570	21,330	20,597	3627	Yes
*Crotaphytus collaris*	Eastern Collared Lizard	2720	1892	606	464	Yes
*Crotaphytus nebrius*	Sonoran Collared Lizard	157	113	66	35	No
*Crotaphytus vestigium*	Baja California Collared Lizard	134	119	28	24	No
*Diadophis punctatus*	Ring‐necked Snake	2870	2206	1140	911	Yes
*Dipsosaurus dorsalis*	Desert Iguana	11,348	10,931	9447	1438	Yes
*Elgaria coerulea*	Northern Alligator Lizard	3805	3260	1835	1478	Yes
*Elgaria kingii*	Madrean Alligator Lizard	670	491	284	203	Yes
*Elgaria multicarinata*	Southern Alligator Lizard	5881	5172	3093	2208	Yes
*Elgaria panamintina*	Panamint Alligator Lizard	67	51	28	21	No
*Gambelia copeii*	Cope's Leopard Lizard	92	67	11	11	No
*Gambelia sila*	Blunt‐nosed Leopard Lizard	620	281	50	44	No
*Gambelia wislizenii*	Long‐nosed Leopard Lizard	30,228	29,531	27,668	5357	Yes
*Gyalopion canum*	Chihuahuan Hook‐nosed Snake	292	165	75	67	Yes
*Gyalopion quadrangulare*	Thornscrub Hook‐nosed Snake	171	155	26	19	No
*Heloderma suspectum*	Gila Monster	2296	2073	1760	755	Yes
*Hemidactylus turcicus*	Mediterranean Gecko	442	300	250	200	Yes
*Heterodon kennerlyi*	Mexican Hog‐nosed Snake	185	176	122	102	Yes
*Heterodon nasicus*	Plains Hog‐nosed Snake	1212	833	476	399	Yes
*Heterodon platirhinos*	Eastern Hog‐nosed Snake	15	1	0	0	No
*Holbrookia elegans*	Elegant Earless Lizard	339	294	123	98	Yes
*Holbrookia lacerata*	Spot‐tailed Earless Lizard	9	1	0	0	No
*Holbrookia maculata*	Common Lesser Earless Lizard	2850	2074	706	451	Yes
*Hypsiglena chlorophaea*	Desert Nightsnake	1273	1177	923	619	Yes^a^
*Hypsiglena jani*	Chihuahuan Nightsnake	321	109	67	65	Yes^a^
*Hypsiglena ochrorhyncha*	Coast Nightsnake	290	197	98	92	Yes^a^
*Hypsiglena torquata*	Night Snake	2243	1659	547	451	Yes^a^
*Lampropeltis alterna*	Gray‐banded Kingsnake	63	8	8	7	No
*Lampropeltis californiae*	California Kingsnake	1000	866	580	533	Yes
*Lampropeltis calligaster*	Prairie Kingsnake	3	0	0	0	No
*Lampropeltis getula*	Eastern Kingsnake	3109	2468	1008	755	Yes
*Lampropeltis holbrooki*	Speckled Kingsnake	3	2	2	1	No
*Lampropeltis knoblochi*	Madrean Mountain Kingsnake	11	11	11	11	No
*Lampropeltis pyromelana*	Arizona Mountain Kingsnake	642	430	265	158	Yes
*Lampropeltis splendida*	Desert Kingsnake	103	92	62	55	Yes
*Lampropeltis triangulum*	Eastern Milksnake	1205	836	417	318	Yes
*Lampropeltis zonata*	California Mountain Kingsnake	847	472	243	195	Yes
*Lichanura orcutti*	Rosy Boa	123	122	104	97	Yes
*Lichanura trivirgata*	Three‐lined Boa	693	562	196	162	Yes
*Micruroides euryxanthus*	Sonoran Coralsnake	541	358	199	115	Yes
*Nerodia erythrogaster*	Plain‐bellied Watersnake	78	51	12	10	No
*Nerodia fasciata*	Southern Watersnake	6	6	5	2	No
*Nerodia rhombifer*	Diamond‐backed Watersnake	11	10	10	9	No
*Nerodia sipedon*	Common Watersnake	76	37	8	8	No
*Opheodrys vernalis*	Smooth Greensnake	400	233	146	118	Yes
*Oxybelis aeneus*	Brown Vinesnake	401	329	143	105	Yes
*Pantherophis emoryi*	Great Plains Ratsnake	232	47	37	36	No
*Petrosaurus mearnsi*	Mearn's Rock Lizard	445	385	102	61	Yes
*Phrynosoma blainvillii*	Blainville's Horned Lizard	2005	1199	538	429	Yes
*Phrynosoma cornutum*	Texas Horned Lizard	2173	1446	765	536	Yes
*Phrynosoma douglasii*	Pygmy Short‐horned Lizard	1526	764	454	341	Yes
*Phrynosoma goodei*	Goode's Horned Lizard	41	31	28	19	No
*Phrynosoma hernandesi*	Greater Short‐horned Lizard	4087	3244	2068	1122	Yes
*Phrynosoma mcallii*	Flat‐tailed Horned Lizard	2056	1594	1410	281	Yes
*Phrynosoma modestum*	Round‐tailed Horned Lizard	1344	983	348	238	Yes
*Phrynosoma platyrhinos*	Desert Horned Lizard	36,111	35,492	33,697	5554	Yes
*Phrynosoma solare*	Regal Horned Lizard	1227	1001	658	322	Yes
*Phyllodactylus nocticolus*	Peninsula Leaf‐toed Gecko	64	58	25	22	No
*Phyllorhynchus browni*	Saddled Leaf‐nosed Snake	387	291	102	70	Yes
*Phyllorhynchus decurtatus*	Spotted Leaf‐nosed Snake	1725	1411	431	334	Yes
*Pituophis catenifer*	Gophersnake	17,968	15,645	10,899	7846	Yes
*Plestiodon callicephalus*	Mountain Skink	141	113	42	22	No
*Plestiodon gilberti*	Gilbert's Skink	1217	1075	339	283	Yes
*Plestiodon multivirgatus*	Many‐lined Skink	398	277	90	69	Yes
*Plestiodon obsoletus*	Great Plains Skink	762	513	199	158	Yes
*Plestiodon septentrionalis*	Prairie Skink	1	0	0	0	No
*Plestiodon skiltonianus*	Western Skink	3505	3135	1802	1269	Yes
*Plestiodon tetragrammus*	Four‐lined Skink	89	22	10	8	No
*Podarcis muralis*	Common Wall Lizard	33	32	32	21	No
*Podarcis sicula*	Italian Wall Lizard	18	18	18	12	No
*Rena dissecta*	New Mexico Threadsnake	64	47	30	25	No
*Rena dulcis*	Texas Threadsnake	176	123	66	58	Yes
*Rena humilis*	Western Threadsnake	880	665	246	183	Yes
*Rhinocheilus lecontei*	Long‐nosed Snake	4190	3245	1560	1130	Yes
*Salvadora grahamiae*	Eastern Patch‐nosed Snake	605	371	187	161	Yes
*Salvadora hexalepis*	Western Patch‐nosed Snake	2790	2196	1195	917	Yes
*Sauromalus ater*	Common Chuckwalla	5997	5733	4806	1203	Yes
*Sceloporus arenicolus*	Dunes Sagebrush Lizard	1254	1192	1147	332	Yes
*Sceloporus bimaculosus*	Twin‐spotted Spiny Lizard	28	24	24	19	No
*Sceloporus clarkii*	Clark's Spiny Lizard	2240	1802	849	423	Yes
*Sceloporus consobrinus*	Prairie Lizard	188	164	49	43	No
*Sceloporus cowlesi*	Southwestern Fence Lizard	489	485	395	275	Yes
*Sceloporus graciosus*	Common Sagebrush Lizard	6196	5017	2912	1909	Yes
*Sceloporus jarrovii*	Yarrow's Spiny Lizard	1884	1552	941	367	Yes
*Sceloporus magister*	Desert Spiny Lizard	4121	3317	1378	809	Yes
*Sceloporus merriami*	Canyon Lizard	260	64	20	17	No
*Sceloporus occidentalis*	Western Fence Lizard	22,822	21,336	16,807	7152	Yes
*Sceloporus orcutti*	Granite Spiny Lizard	950	831	307	184	Yes
*Sceloporus poinsettii*	Crevice Spiny Lizard	999	681	211	158	Yes
*Sceloporus scalaris*	Bunch Grass Lizard	86	83	30	19	No
*Sceloporus slevini*	Slevin's Bunchgrass Lizard	219	200	149	81	Yes
*Sceloporus tristichus*	Plateau Fence Lizard	925	579	284	230	Yes
*Sceloporus undulatus*	Eastern Fence Lizard	3907	3019	893	627	Yes
*Sceloporus uniformis*	Yellow‐backed Spiny Lizard	9697	9694	9694	2497	Yes
*Sceloporus virgatus*	Striped Plateau Lizard	520	437	258	108	Yes
*Senticolis triaspis*	Green Ratsnake	443	381	178	114	Yes
*Sistrurus catenatus*	Eastern Massasauga	228	165	103	81	Yes
*Sistrurus miliarius*	Pygmy Rattlesnake	2	2	0	0	No
*Sonora semiannulata*	Western Groundsnake	1353	889	380	292	Yes
*Storeria dekayi*	Dekay's Brownsnake	103	40	16	16	No
*Storeria occipitomaculata*	Red‐bellied Snake	57	24	16	16	No
*Tantilla atriceps*	Mexican Black‐headed Snake	29	26	4	4	No
*Tantilla hobartsmithi*	Smith's Black‐headed Snake	588	365	216	110	Yes
*Tantilla nigriceps*	Plains Black‐headed Snake	567	354	145	124	Yes
*Tantilla planiceps*	Western Black‐headed Snake	310	236	59	55	Yes
*Tantilla wilcoxi*	Chihuahuan Black‐headed Snake	63	53	20	17	No
*Tantilla yaquia*	Yaqui Black‐headed Snake	91	73	41	30	No
*Thamnophis atratus*	Aquatic Gartersnake	1324	1115	649	479	Yes
*Thamnophis couchii*	Sierra Gartersnake	1132	818	385	290	Yes
*Thamnophis cyrtopsis*	Black‐necked Gartersnake	1779	1344	700	484	Yes
*Thamnophis elegans*	Terrestrial Gartersnake	12,674	10,590	6684	4826	Yes
*Thamnophis eques*	Mexican Gartersnake	702	607	342	180	Yes
*Thamnophis gigas*	Giant Gartersnake	411	50	16	16	No
*Thamnophis hammondii*	Two‐striped Gartersnake	941	638	235	206	Yes
*Thamnophis marcianus*	Checkered Gartersnake	1304	837	401	265	Yes
*Thamnophis ordinoides*	Northwestern Gartersnake	2489	1872	801	576	Yes
*Thamnophis proximus*	Western Ribbonsnake	478	294	110	97	Yes
*Thamnophis radix*	Plains Gartersnake	2749	2379	1971	1608	Yes
*Thamnophis rufipunctatus*	Narrow‐headed Gartersnake	840	785	671	175	Yes
*Thamnophis sirtalis*	Common Gartersnake	8146	6906	4436	3149	Yes
*Thamnophis* sp.	Gartersnake, Ribbonsnake	52	43	43	34	No
*Trimorphodon biscutatus*	Western Lyresnake	1089	835	238	186	Yes
*Trimorphodon lambda*	Sonoran Lyresnake	540	534	494	121	Yes
*Trimorphodon lyrophanes*	California Lyresnake	23	21	5	5	No
*Trimorphodon vilkinsonii*	Texas Lyre Snake	55	5	4	4	No
*Tropidoclonion lineatum*	Lined Snake	133	81	45	45	No
*Uma inornata*	Coachella Fringed‐toed Lizard	374	171	28	28	No
*Uma notata*	Colorado Desert Fringe‐toed Lizard	420	312	58	43	No
*Uma rufopunctata*	Yuman Desert Fringe‐toed Lizard	48	48	30	27	No
*Uma scoparia*	Mohave Fringe‐toed Lizard	424	315	146	73	Yes
*Urosaurus graciosus*	Long‐tailed Brush Lizard	857	670	263	202	Yes
*Urosaurus nigricaudus*	Baja California Brush Lizard	774	681	151	139	Yes
*Urosaurus ornatus*	Ornate Tree Lizard	4461	3299	1565	927	Yes
*Uta stansburiana*	Common Side‐blotched Lizard	18,102	15,887	8984	4834	Yes
*Xantusia arizonae*	Arizona Night Lizard	52	41	9	8	No
*Xantusia bezyi*	Bezy's Night Lizard	20	19	17	10	No
*Xantusia gracilis*	Sandstone Night Lizard	6	5	4	3	No
*Xantusia henshawi*	Granite Night Lizard	513	428	88	71	Yes
*Xantusia riversiana*	Island Night Lizard	185	126	21	12	No
*Xantusia vigilis*	Desert Night Lizard	1777	1467	604	413	Yes
*Xantusia wigginsi*	Wiggins' Night Lizard	137	131	31	29	No
		427,846	367,953	257,427	110,121	

^a^ The species was modeled at the genus level.

## Appendix 3

Climate variables used in the species climate‐niche modeling. These variables were derived from the ClimateNA v5.5 tool (Wang et al. 2016). Variables were generated for each period and climate projection scenario: past (1901–1930), recent (1981–2010), future Representative Concentration Pathway (RCP) 4.5 (2040–2069 and 2070–2100), and future RCP 8.5 (2040–2069 and 2070–2100).

**Table:**

Acronym	Climate variable	Definition
PPT01–PPT12	Monthly precipitation	The average total precipitation for each of the months January–December (mm)
Tave01–Tave12	Monthly average temperature	The average mean temperature for each of the months January–December (°C)

## Appendix 4

True skill statistic (TSS) score summaries for eight modeling techniques across all squamate species (*n* = 130) and runs (four per species; Jeffries et al. 2024a, 2024b).

**Table:**

Model	Mean	Standard deviation	Median	Min	Max	Count non‐converged	Count converged
ANN	0.84	0.09	0.85	0.49	0.99	2	518
CTA	0.87	0.07	0.88	0.55	0.99	0	520
FDA	0.84	0.10	0.85	0.001	0.99	142	378
GAM	0.84	0.10	0.86	0.23	0.99	16	504
GLM	0.85	0.11	0.87	0.002	0.99	1	519
MARS	0.88	0.09	0.89	0.07	0.99	0	520
RF	0.89	0.07	0.90	0.64	0.99	0	520
SRE	0.65	0.09	0.66	0.27	0.90	0	520

Abbreviations: ANN, Artificial neural network; CTA, Classification tree analysis; FDA, Flexible discriminant analysis; GAM, Generalized additive model; GLM, Generalized linear model; MARS, Multiple adaptive regression splines; RF, Random forest; SRE, Surface range envelope.

## Appendix 5

Receiver operating characteristic (ROC) and true skill statistic (TSS) summary across all modeled species (Jeffries et al. 2024a, 2024b).

**Table:**

Evaluation statistic	Mean evaluation score	Evaluation range	Mean sensitivity	Sensitivity range	Mean specificity	Specificity range
ROC	0.99	0.94–1.00	98.4	90.4–100	95.4	85.9–99.9
TSS	0.94	0.77–0.99	98.4	90.4–100	95.4	85.9–99.9

## Appendix 6

Climate variable importance summary across all modeled species. Months are depicted as 01–12 on *x*‐axis climate metric acronyms (Appendix 2: PPT = average monthly precipitation; *T*_ave_ = average monthly average temperature), with 25th and 75th percentiles, a median line, whiskers extending to 1.5‐times the interquartile range, and outliers displayed as points (Jeffries et al. 2024a, 2024b).
